# ‘ILC-poiesis’: generating tissue ILCs from naïve precursors

**DOI:** 10.18632/oncotarget.21046

**Published:** 2017-09-19

**Authors:** James P. Di Santo, Ai Ing Lim, Hans Yssel

**Affiliations:** James P. Di Santo: Innate Immunity Unit, Institut Pasteur, Inserm U1223, Paris, France

**Keywords:** innate lymphoid cells

Innate lymphoid cells (ILC) are a newly described family of hematopoietic cells that lack antigen-specific receptors but can be activated to promptly produce large amounts of cytokines (including interleukin (IL)-5, -13, -17A, -22, TNF-α and interferon-γ) and thereby contribute to the immediate, first-line immune defense against viral, bacterial, and parasitic infections (reviewed in [1]). ILCs include the previously described natural killer (NK) cells and have a similar natural effector function which is immediately available during immune responses and prior to that of adaptive immunity. Three groups of ILC (ILC1, ILC2, ILC3) have been described, that along with NK cells, share biological activities of T helper (Th)1, Th2 and Th17/22 subsets and CTL, respectively. ILCs are active during both fetal and adult life and play important roles in the homeostasis of mucosal and non-mucosal tissues. How ILCs develop from hematopoietic precursors is poorly defined and how ILCs are integrated into ongoing immune responses remains unclear. Knowledge in this arena is a prerequisite for harnessing the clinical potential of these potent immune effector cells.

In a recent report [2], we identified a subset of circulating and tissue-resident human ILCs (lineage^-^CD7^+^CD127^+^CD56^-^ cells) that represent *bona fide* ILC precursors (ILCP). Human CD117^+^ ILCP were shown to give rise to the three well characterized helper ILC subsets (ILC1, ILC2, ILC3) as well as cytotoxic NK cells *in vitro* and *in vivo* using humanized mice [2]. Human ILCP were a heterogeneous population comprising uni-potent (able to generate one ILC subset) and multi-potent (able to generate two or more ILC subsets) precursors. This observation was possible thanks to development of a robust single cell cloning assay [3] that took lessons from earlier T cell cloning methods [4]. Human ILCP were shown to express transcription factors (TF) known to control ILC differentiation in the mouse [5] but did not express transcripts for signature TF (T-BET, GATA-3, RORC) or cytokines (IL-5, -13, -17A, -22 or IFN-γ) found in mature tissue-resident ILCs. Epigenetic analysis showed that human ILCP harbored these genes in a poised state that could be activated given the proper environmental signal. Along these lines, we found that IL-1β was a critical factor for generation of mature ILC and NK cells from human ILCP [2]. Accordingly, we coined the term ‘ILC-poiesis’ to describe the process whereby ILCP circulate throughout the organism (via the blood) and can be activated in tissues by local inflammatory signals that trigger their appropriate differentiation into mature ILCs and/or NK cells [2]. Our ILC-poiesis model challenges the existing dogma that the bone marrow is the central factory for generating tissue ILCs and proposes a local site of ILC production in tissues.

**Figure 1:**
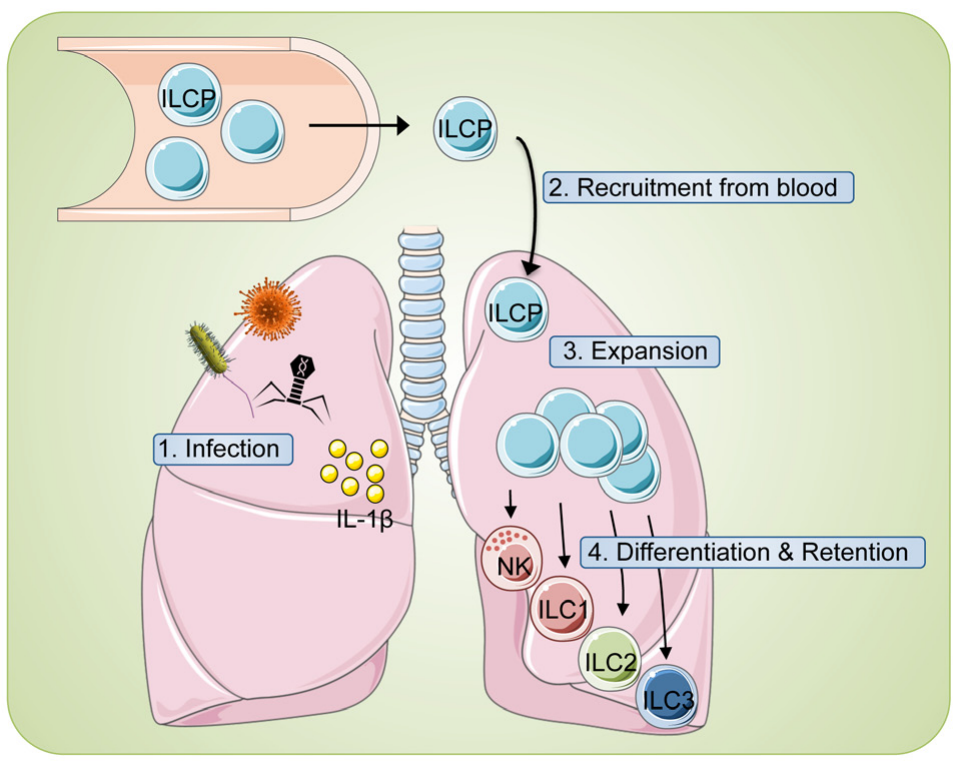
‘ILC-poiesis’ allows for the generation of tissue ILCs ‘on demand’ 1) Local tissue disturbances (infection, inflammation, etc.) are often associated with inflammasome activation and liberation of IL-1β (yellow circles). 2) Circulating ILC precursors (ILCP) are subsequently recruited from blood and 3) stimulated to expand in response to IL-1β. 4) Additional tissue signals then guide appropriate ILC differentiation to combat the inciting event and also result in ILC tissue retention.

Comparing ILC-poiesis with the current model of T cell differentiation reveals several thought-provoking parallels. Functionally diverse T cell subsets derive from naïve T cells that encounter antigenic stimulation, co-stimulation and polarizing cytokines from dendritic cells within secondary lymphoid tissues (reviewed in [6]). In ILC-poiesis, ILCP may represent the functional equivalent of naïve ILCs that can similarly provide the cellular substrate for mature tissue-resident ILCs. Human ILCP are small, resting lymphocytes that are homogeneously CD45RA^+^, CD69^-^ and CD62L^+^, similar to naïve T cells. Upon encounter with inflammatory signals (such as IL-1β), ILCP proliferate and differentiate; this trigger may represent the innate equivalent of the antigen-receptor pathway that strongly activates NF-kB. Additional roles for co-stimulation and cytokines are also likely and may vary dependent on the environmental context (Figure 1). Taken together, our results suggest that the cellular mechanisms controlling innate (ILC) and adaptive (T cell) differentiation share striking similarities and likely proceed in an analogous fashion, in contrast to the prevailing notion that ILCs and NK cells are born as ‘hard-wired’ effector cells that are ‘ready to go’.

Over 10 years ago, we had proposed that circulating NK cell restricted precursors might allow for the generation of NK cells in peripheral tissues [7]. Our current model of ILC-poiesis extends this concept and proposes that any tissue can become a rapid, on-demand factory for ILC development. As such, inflamed tissues are the functional equivalent of secondary lymphoid tissues that drive adaptive T cell differentiation. The signals that attract circulating ILCP into inflamed tissues remain to be defined, as well as the APC equivalent that provides IL-1β and other cytokine signals for ILC differentiation. It is also possible that ILC differentiation occurs within secondary lymphoid tissues concurrently with classical T cell differentiation. While this process would not have the rapid kinetics of tissue-intrinsic ILC differentiation, it could provide an interesting (and reinforcing?) second wave of ILCs in the context of inflammation or infection.
